# TLR9 is required in donor blood for inflammation-induced immunization to an alloantigen on transfused RBCs in mice

**DOI:** 10.3389/fimmu.2026.1952683

**Published:** 2026-10-26

**Authors:** Arijita Jash, Krystalyn E. Hudson, James C. Zimring

**Affiliations:** 1 Beirne Carter Immunology Center, University of Virginia, Charlottesville, VA, United States; 2 Department of Pathology, University of Virginia, Charlottesville, VA, United States; 3 Department of Pathology and Cell Biology, Columbia University, New York, NY, United States; 4 Canadian Blood Services, Ottawa, ON, Canada; 5 Department of Pathology and Laboratory Medicine, University of British Columbia, Vancouver, BC, Canada

**Keywords:** alloimmnuzation, CpG, RBC (red blood cell), toll like receptor, transfusion

## Abstract

Humoral immunization to alloantigens on transfused red blood cells (RBCs) remains a major barrier to safe and effective blood transfusion in chronically transfused patients. However, only some patients become alloimmunized following multiple transfusion. It was first observed in mice and then confirmed in humans that recipient inflammation at the time of transfusion increases rates of RBC alloimmunization. Viral double-stranded RNA has been extensively studied and shown to enhance alloimmunization through activation of recipient MAVS (mitochondrial antiviral-signaling protein) and subsequent induction of alpha-interferon. However, no one has reported testing the role of innate immune receptors on donor cells. Herein, we report that recipient exposure to another innate immune activator (CpG) enhances alloimmunization to transfused RBCs in a TLR9-dependent fashion. In addition, TLR9 is required both on the transfused donor cells and in the recipient. To the best of our knowledge, this is the first report of innate immune receptors on transfused donor cells regulating RBC alloimmunization. In addition to adding a new conceptual component to the mechanistic landscape of RBC alloimmunization, these findings raise the possibility of decreasing RBC alloimmunization either by selecting RBC units from donors with known hypomorphic TLR9 gene variants or by introducing TLR9 antagonists into donor blood.

## Introduction

Humoral immunization to alloantigens on red blood cells (RBC alloimmunization) remains a significant medical problem in transfusion medicine (1, 2). While some patients become progressively immunized to multiple alloantigens, others receive hundreds of transfusions without mounting a single detectable alloantibody (3). The basis for this “responder” vs. “non-responder” pattern remains only partially understood and has been identified as a research priority by the field (4). Mechanistic understanding of causative factors that regulate RBC alloimmunization would provide immediate benefit in managing specific patient transfusion and also serve as a rational basis for developing therapeutic interventions.

Recipient inflammation at the time of transfusion is a major regulator of RBC alloimmunization in both humans and mouse models (5–7). Inflammation and innate immune activation are induced by chemical motifs found in microbes but not in mammals [i.e., pathogen-associated molecular patterns (PAMPS)] (8). Two widely studied PAMPS are double-stranded RNA (dsRNA) and CpG, which are found in the life cycle of viruses and bacteria, respectively. Using a murine model of transfusion, we first reported that recipient inflammation at the time of transfusion is a major regulator of RBC alloimmunization, a finding that has now translated into humans (5–7). Antibody responses are significantly increased by recipient exposure to dsRNA or influenza virus before transfusion in multiple murine alloantigen models (i.e., mHEL, HOD, KEL1, KEL2, and hGPA) by signaling through MAVS (mitochondrial antiviral-signaling protein) followed by induction of type I interferons that directly regulate T- and B-cell function through the interferon-alpha/beta receptor (IFNAR1) (5, 9, 10). Viremia at the time of transfusion is also associated with increased alloimmunization in humans (6, 11).

We further reported that RBC alloimmunization is differentially regulated by distinct regulators of innate immunity in mice (12). Exposure of recipient mice to dsRNA found in the life cycle of many viruses increases RBC alloimmunization while lipopolysaccharide (LPS) found in gram-negative bacteria decreases RBC alloimmunization (12). Human studies have confirmed the same pattern with viremia at the time of transfusion correlating with increased RBC alloimmunization and gram-negative bacteremia correlating with decreased RBC alloimmunization (6). Yu et al. have also shown that DNA motifs found in multiple gram-negative and gram-positive bacteria (i.e., CpG) also enhance RBC alloimmunization to murine RBCs expressing human glycophorin A (hGPA) (13).

Mangalmurti et al. have recently reported that TLR9 is expressed on human and murine RBCs and that TLR9 on murine RBCs affects innate immunity in response to CpG (14). TLR9 is the primary receptor for CpG in mammals but has not been assessed in CpG-induced RBC alloimmunization. We thus hypothesized that TLR9 on donor RBCs affected adaptive humoral alloimmunization. We report that CpG-induced alloimmunization requires TLR9 expression both on donor blood and in recipient mice. A model is presented consistent with known immunology and each of the findings reported herein.

## Methods

*Mice:* HOD mice were generated as previously described on a C57BL/6 background (15). HOD mice express a triple fusion protein made from hen egg lysozyme (HEL) fused to the C-terminal half of ovalbumin (OVA) fused to human Duffy (HEL-OVA-Duffy = HOD). The HOD mice used were the variant with a single copy of HOD knocked into the ROSA-26 locus (15). TLR9^−/−^ mice (Cat#034449) and wild-type B6 mice were each purchased from The Jackson Laboratory (Bar Harbor, ME, USA). HOD mice were bred with TLR9^−/−^ mice to generate HODXTLR9^−/−^ mice. Expression of TLR9 and HOD on RBCs was confirmed by staining with anti-TLR9 (BioLegend 159105) and anti-HEL (4B7) or anti-Ova (Puma6) antibodies, respectively (16, 17). All recipient mice were female, 8–12 weeks of age, and all procedures were approved by the Animal Care and Use Committee of the University of Virginia (Protocol Title: Immunology of RBC Transfusion, Protocol number: 4588).

*Transfusion and treatment with CpG:* RBC donor whole blood was collected and transfused precisely as previously described (18). Briefly RBC donor blood was collected in citrate phosphate dextrose adenine 1 (CPDA-1) and leukoreduced by aspirating the RBC pellet after centrifugation to obtain packed RBC. Fifty microliters of packed RBCs representing the physiological equivalent of a standard 1-unit human RBC transfusion was transfused intravenously into mice that previously received an injection of either phosphate-buffered saline (PBS) or 50 μg of CpG (InvivoGen Cat# ODN1856) dissolved in PBS intraperitoneally 2 h before transfusion (13, 19).

*Alloantibody measurement:* Anti-HOD alloantibodies were measured using a flow cytometry-based cross-match as previously described (20). Briefly, sera from transfused mice were collected at the indicated time points and incubated with target RBCs expressing either HOD (antigen^+^ RBCs) or B6 (antigen^–^ RBCs) followed by the fluorescently labeled secondary antibody. The antigen-specific response (i.e., adjusted MFI) was determined by subtracting the MFI of B6 RBCs (background) from the MFI of HOD RBC targets. All flow cytometry was performed on an Attune NxT Cytometer (Thermo Fisher Scientific) and analyzed using FlowJo software.

*Statistical analysis:* Mann–Whitney test (**Figures 1B, C**), Kruskal–Wallis test (**Figures 1D, E**), mixed-model analysis of variance (ANOVA) with Sidak’s multiple comparison (**Figures 2D, E**), and, to control variation between experiments for pooled analysis sample containing multiple experiments, linear mixed-model effect (**Figure 2F**) were used for statistical analysis with *p* < 0.05 being significant. All data are presented as the mean ± SD.

**Figure 1:**
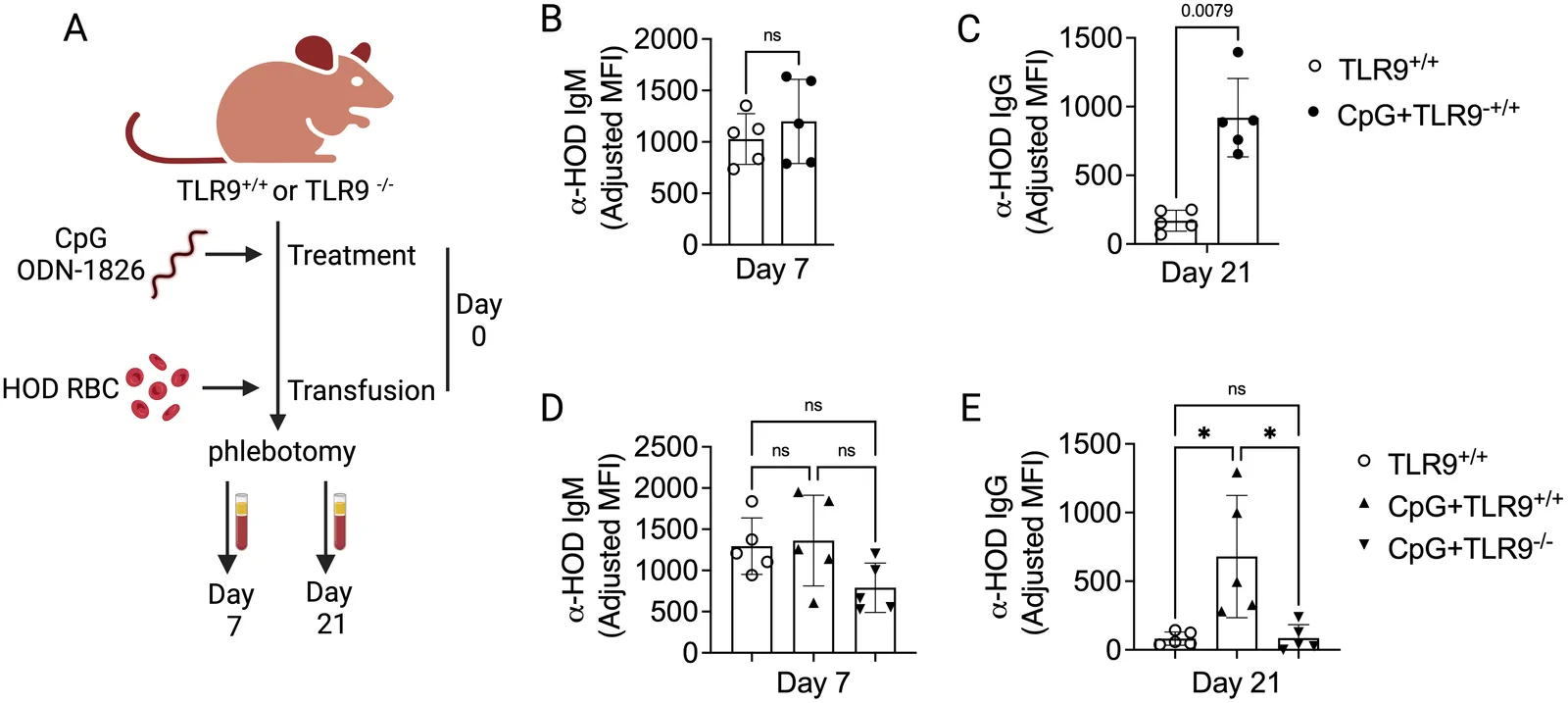
Alloimmune response to HOD RBCs is independent of TLR9 expression on recipient RBCs but not from other cell types. **(A)**. General experimental scheme. **(B–E)** Mice were bled at the indicated time points and the amount of anti-HOD IgM on day 7 and anti-HOD IgG was measured by a flow cytometry-based cross-match assay using either HOD or B6 RBCs as targets as detailed in the Methods section. Adjusted MFIs are calculated by subtracting the background antibody signal on antigen-negative B6 RBCs from RBCs expressing HOD. Representative experiments with three repeats with similar outcomes with five mice per group are shown. *p*-values are calculated using a Kruskal–Wallis test and *p*-values of less than 0.05 were considered statistically significant. All data are presented as the mean ± SD. *P* > 0.05 (NS) and *P < 0.05, **P < 0.01, and ***P < 0.0005. Created with BioRender.com.

**Figure 2:**
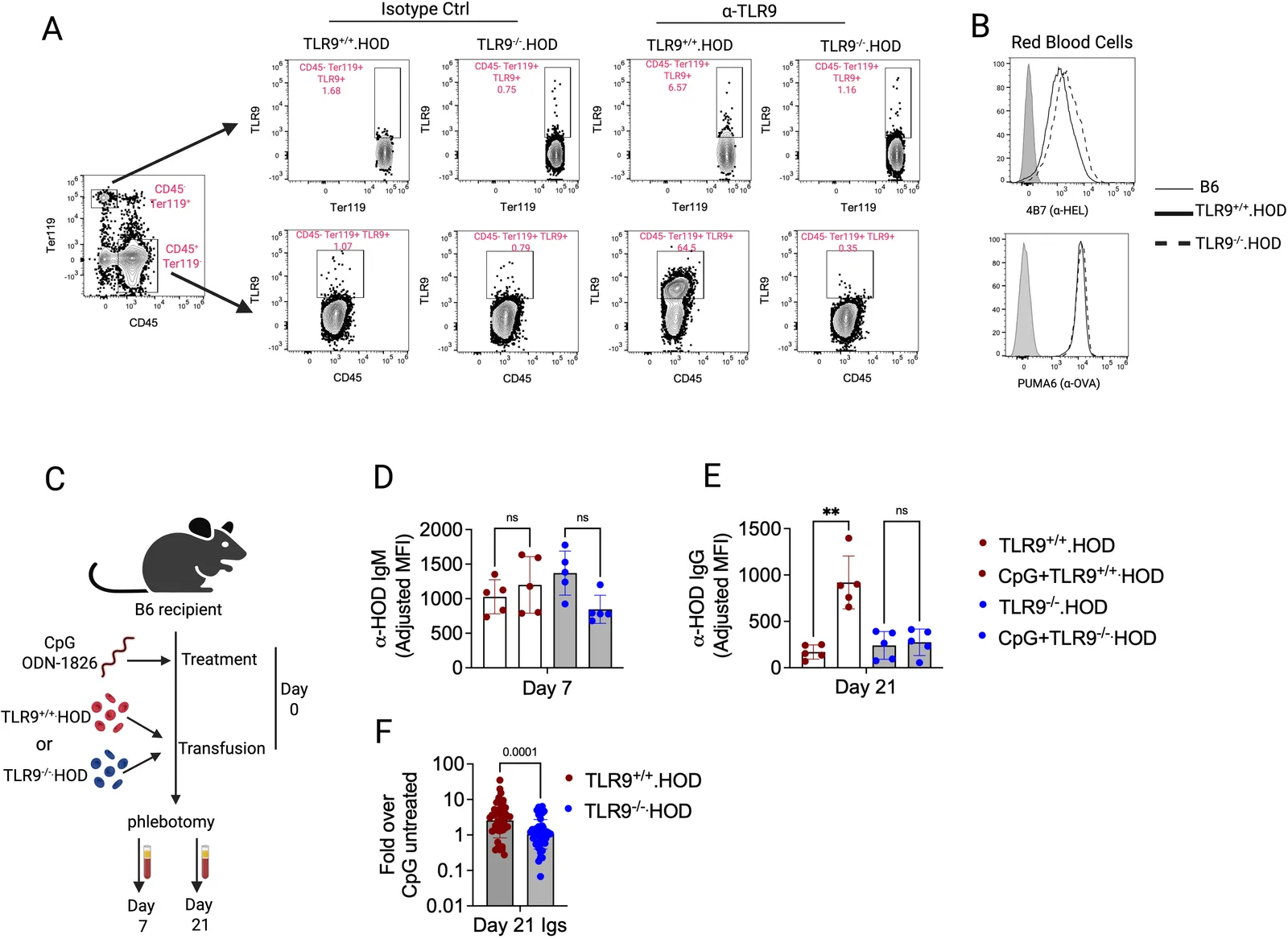
Alloimmune response to HOD RBCs is dependent on TLR9 expression on donor RBCs. **(A, B)** Expression of HOD and TLR9 in HOD.TLR9^+/+^ and HOD.TLR9^−/−^ in the donor RBCs was confirmed by staining with anti-HOD antibodies [4B7 (anti-HEL) and PUMA6 (ant-OVA)]. **(C)** General experimental scheme. **(D, E)** Mice were bled at the indicated time points and the amount of anti-HOD IgM on day 7 and anti-HOD IgG was measured by a flow cytometry-based cross-match assay using either HOD or B6 RBCs as targets as detailed in the Methods section. Adjusted MFIs are calculated by subtracting the background antibody signal on antigen-negative B6 RBCs from RBCs expressing HOD. Representative experiments with seven repeats with 5–10 mice per group are shown. **(F)** Fold response to CpG treatment over the mean of PBS-treated mice was calculated [fold response = *m*_CpG_/mean (*m*_PBS_), where *m* = individual mouse] and fold over PBS for each transfusion group (HOD.TLR9^+/+^ vs. HOD.TLR9^−/−^) and data are represented as a pool of seven independent experiments. *p*-values are calculated using a mixed-model analysis with Sidak’s multiple comparison, Mann–Whitney test, and a linear mixed-effects model to control for experiment-to-experiment variability. *P*- values of less than 0.05 were considered statistically significant such as *P* > 0.05 (NS) and *P < 0.05, **P < 0.01, and ***P < 0.0005. All data are presented as the mean ± SD. Created with BioRender.com.

## Results and discussion

In this study, we utilized the widely used HOD mice that express an RBC-specific model antigen consisting of HEL fused to the C-terminal half of OVA fused to human Duffy (21). HOD RBCs were transfused into recipients pre-treated with 50 μg of CpG or diluent control PBS 2 h before transfusion; anti-HOD alloantibodies were measured at 7 and 21 days post-transfusion by flow cross-match using established methods (20) (**Figure 1A**). No significant change in anti-HOD IgM on day 7 was observed in response to CpG (**Figure 1B**). CpG pre-treatment caused a 5.4-fold increase in anti-HOD IgG compared to control mice on day 21 (*p* = 0.01) (**Figure 1C**). The best-known receptor for CpG is TLR-9. While CpG did not significantly alter the anti-HOD IgM response (**Figure 1D**), its ability to enhance anti-HOD IgG alloantibodies was completely abolished in TLR9^−/−^ recipient mice (**Figure 1E**).

To the best of our knowledge, this is the first demonstration of CpG-induced alloimmunization to an alloantigen other than hGPA, suggesting that CpG enhances RBC alloimmunization in general.

To date, all studies of innate immune receptor regulation of RBC alloimmunization have focused exclusively on recipient cells. However, Lam et al. recently reported that both human and murine RBCs express TLR9 (14). To test the role of TLR9 in RBC alloimmunization, HOD mice were crossed with TLR9 knockout mice (HODxTLR9^−/−^). Flow cytometric staining with anti-TLR9 confirmed TLR-9 expression in RBCs, which was eliminated in HOD^+^TLR9^−/−^ RBCs (**Figure 2A**). Deletion of TLR9 had no effect on HOD antigen expression on RBCs (**Figure 2B**). Wild-type B6 recipients were treated with CpG and then transfused with blood from either HOD or TLR9^−/−^.HOD donors (**Figure 2C**). While no significant difference was observed in day 7 IgM response (**Figure 2D**), CpG-induced anti-HOD IgG responses to HOD were significantly reduced in wild-type recipients transfused with HOD^+^TLR9^−/−^ RBCs compared to HOD^+^TLR9^+/+^ on day 21 (**Figure 2E**). Anti-HOD IgG responses on day 21 were calculated as fold increase of CpG-induced responses over PBS control to enable combination of multiple experiments. The combined results of seven different experiments confirmed that anti-HOD IgG responses to HOD^+^TLR9^−/−^ donor blood were significantly lower than HOD^+^TLR9^+/+^ blood (*p* = 0.0001) (**Figure 2F**).

The data herein are the first identification of TLR9 as a response element in CpG-induced RBC alloimmunization. This result may seem obvious because dsRNA and CpG are well-known agonists of TLR-3 and TLR-9, respectively (22). Indeed, based on this background knowledge, some have even stated that the mechanism of dsRNA and CpG-induced RBC alloimmunization is through TLR-3 and TLR-9, respectively (23). However, this supposition has been shown to be incorrect for dsRNA, which does not require TLR-3 for enhancement of RBC alloimmunization. Rather, dsRNA-induced RBC alloimmunization works through an alternate dsRNA-activated pathway involving MAVS and induction of IFN*-α*/β with subsequent effects on antigen-presenting cells (APCs) and B cells through IFNAR (9). Like dsRNA, CpG can signal through non-TLR pathways (i.e., cGAS/STING); however, the current findings identify TLR9 as being required for RBC alloimmunization (24).

In contrast, the requirement for TLR9 expression in donor blood was an unanticipated finding that expands the current paradigm of innate immune regulation in RBC alloimmunization to focus on donor as well as recipient biology. While TLR9 is expressed on both human and murine RBCs, the exact molecular mechanism linking erythrocyte-bound CpG to subsequent lymphocyte activation remains a working hypothesis. The physical mechanisms by which RBC-intrinsic TLR9 cooperates with B-cell receptor (BCR) engagement or plasmacytoid dendritic cell (pDC) uptake through endocytic co-engagement or localized antigen presentation warrant direct empirical testing. A comprehensive experimental dissection of these signaling cascades exceeds the primary scope of this Brief Report, which is focused on establishing the novel genetic and cellular requirement of donor-intrinsic TLR9 in RBC alloimmunization; however, it remains the focus of our active ongoing investigations. Nevertheless, we consider it appropriate to posit several hypotheses that are consistent with known immunology (**Figure 3**). Full activation and differentiation of B cells requires simultaneous activation of the cell surface BCR and TLR9 in endosomes (25). Importantly, B cells specifically endocytose antigens for which their BCR is specific. Physical association of CpG with the recognized antigen synchronizes BCR and TLR9 activation. When a B cell has a BCR specific for an RBC surface antigen, it is not presently known how much of the RBC is endocytosed (26). However, linked recognition studies in mice support the notion that the B cell endocytoses both the membrane material and cytosolic content of the RBC (26). As such, we propose a model by which TLR9 on the donor RBCs associates CpG with alloantigen recognized by B cells, thereby providing the simultaneous activation of TLR9 and BCR in alloantigen-specific B cells (**Figure 3A**). An alternative hypothesis is that pDCs, which internally express TLR9, are activated upon erythrophagocytosis of RBCs bound with CpG (27). In turn, pDCs present alloantigen-derived peptides in MHC-II, along with co-stimulation and proinflammatory cytokines, to stimulate CD4^+^ T-cell activation (**Figure 3B**); activated pDCs can also enhance B-cell activation through cell-to-cell contact or native protein on the pDC surface (**Figure 3A**). However, we do not favor this hypothesis as it is unclear why TLR9 on donor blood would be required for DC consumption of CpG that is given systemically to recipient mice. We have reported that increased RBC clearance can cause increased alloimmunization, and Lam et al. reported that deletion of TLR9 on RBCs decreases circulation (14, 28). Thus, a third hypothesis is that TLR9 expression on RBCs is facilitating increased erythrophagocytosis into immunogenic APCs. Increased clearance of transfused RBCs is associated with higher alloimmunization rates in mice (28). Assessment of RBC survival and rate of erythrophagocytosis of HOD^+^TLR9^−/−^ versus HOD^+^TLR9^+/+^ RBCs in CpG-treated recipients will be required to definitively separate clearance-dependent antigen presentation effects from direct signaling events. However, since TLR9^−/−^ RBCs have decreased RBC alloimmunization, this line of thinking predicts the opposite of what was observed. Thus, formal *in vivo* clearance half-life measurements remain an important next step.

**Figure 3:**
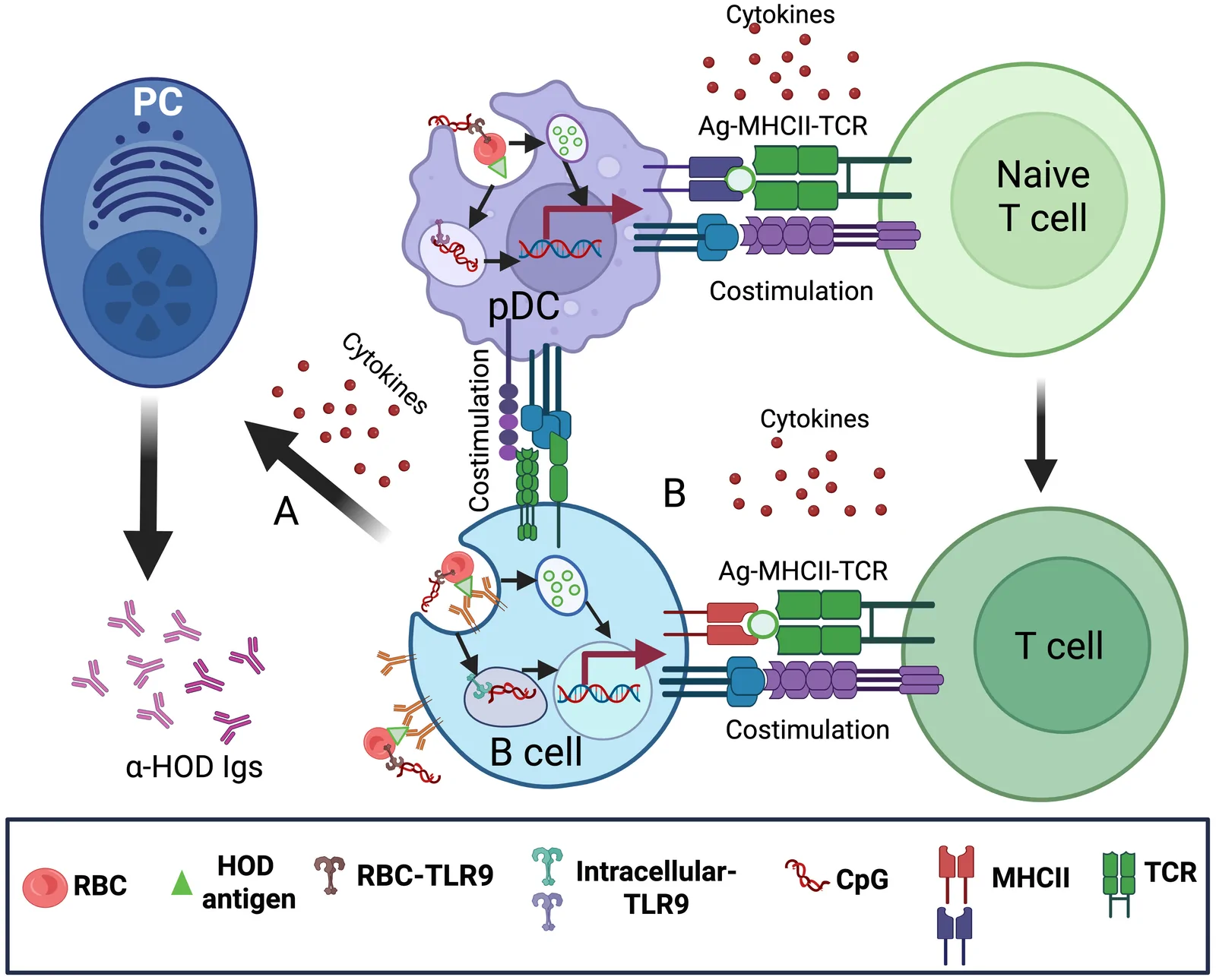
Proposed model depicting the requirement TLR9 on donor RBC and recipient immune cells (B cells) for enhanced alloresponse to HOD RBCs. **(A)** BCR-mediated endocytosis of CpG-bound allogeneic RBCs translocates the CpG to the TLR9 located in the B-cell endosomal compartment. Simultaneous activation of the BCR and TLR9 results in B-cell proliferation and differentiation to anti-HOD antibody-secreting plasma cells. In parallel, plasmacytoid dendritic (pDC) cells via erythrophagocytosis uptake allogenic RBC-bound CpG that activates pDCs by binding to TLR9 located internally in the pDCs. Activated pDCs enhance B-cell proliferation and differentiation to anti-HOD Ig-secreting plasma cells both via direct cell-to-cell contact and via cytokine secretion. **(B)** Activated mature pDCs following CpG-RBC endocytosis and TLR9 binding present processed allogenic RBC peptide–MHC-II complex to naïve T cells resulting in T-cell activation. Activated T cells recognize B cells that process and present the same RBC allopeptide–MHC-II complex along with co-stimulation resulting in differentiation of B cells into anti-HOD Ig-secreting plasma cells. Created with BioRender.com.

None of the above hypotheses are mutually exclusive, and future studies should assess them. While the current manuscript establishes a link between donor TLR9 and RBC alloimmunization, the precise molecular mechanism linking erythrocyte-bound CpG to lymphocyte priming remains a working model. Thus, unraveling whether this operates via direct B-cell endocytic co-engagement of cognate antigen and TLR9 (**Figure 3A**) versus indirect transfer to pDCs (**Figure 3B**) will require future experimental efforts.

As with all studies, the current report has limitations. Because HOD^+^TLR9^−/−^ mice were generated from global TLR9 knockout animals and were not an RBC-specific conditional deletion, we cannot assess the extent to which TLR9 expression in RBCs versus other cell types is required. We consider TLR9 expression on RBCs to be most likely involved, as the HOD antigen is RBC-specific and all mice are on a B6 background; thus, all transfused leukocytes or platelets represent a cell type already present in the recipient. Nevertheless, a finer assessment of donor leukocytes or platelets should be included in ongoing studies.

The current findings are significant at several levels. Considerable progress has been made in identifying how innate immune pathways in transfusion recipients regulate RBC alloimmunization, and the role of damage-associated molecular patterns (DAMPs) on donor RBCs is also being investigated. However, to the best of our knowledge, no one has considered the role of innate immune receptors in donor RBCs. Thus, the current findings expand the paradigm of RBC alloimmunization to include immune regulatory gene products in donor cells and may also have relevance to mechanisms of maternal immunization to fetal RBCs. Although TLR9 signaling is conserved between humans and mice, with similar downstream effects, there are also differences; as with all mouse studies, future translational work will assess whether these findings can be extended to humans (29, 30). Whether donor RBC TLR9 phenotype modulates alloimmunization risk in human cohorts is currently unknown. Should these findings translate into practice, however, the practical ramifications would be substantial for several reasons (31–33). First, there are TLR9 polymorphisms in human donors that may allow selection of less immunogenic RBCs for use in patients who can be harmed by alloimmunization [e.g., patients with sickle cell disease (SCD), myelodysplastic syndromes (MDS), etc.] (34–36). The feasibility of such an approach requires that there is variant TLR9 expression in human RBCs, which has not been tested to the best of our knowledge. Second, significant advances are being made to allow generation of cultured RBCs for patient populations (37). The stem cells used to generate these RBCs are being modified to remove alloantigen (38). In this same vein, purposeful manipulation of innate immune receptors in RBCs may generate less immunogenic units. Finally, there are specific TLR9 antagonists that could, in theory, be added to blood prior to transfusion. In summary, while our findings establish a novel requirement for TLR9 expression in donor blood during CpG-induced RBC alloimmunization, limitations still remain. Since, in our model, the HOD antigen is specifically expressed on RBCs, the effect of CpG on the anti-HOD response strongly indicates a role of TLR9 expressed by RBCs during alloimmunization. However, because we utilized donor blood from germline TLR9 knockout mice rather than erythroid lineage or RBC-specific conditional TLR9 knockout mice, all transfused donor cell types lacked TLR9. Consequently, we cannot definitively exclude contributions from minor donor leukocyte or platelet populations. Ongoing studies utilizing cell-type-specific TLR9 deletions will further resolve these compartment-specific contributions. Conceptually, our study shifts the focus of transfusion-induced alloimmune response from recipients to donor. Mechanistically, understanding how RBC TLR9 ligand binding is associated with B-cell or dendritic cell activation provides a new foundation for mitigating alloimmunization risks in chronically transfused cohorts.

## Data Availability

The raw data supporting the conclusions of this article will be made available by the authors, without undue reservation.
